# Senile Gangrene

**Published:** 1868-03-15

**Authors:** James T. Newman


### SENILE GANGRENE.

BY JAMES T. NEWMAN, M.D.

I WAS called, the 3rd of December, 1867, to see an old lady, 99 years of age. She was suffering with gangrena senilis. On inquiring into the history of her case, I found that she had valvular disease of the heart, and that she had been laboring under the difficulty about twenty years. I also discovered that seven years ago she had an erysipelatous affection. She recovered from the last named disease, and enjoyed good health, until she contracted dry mortification of the right foot. The course of this terrible malady is too well known to require a minute description. Suffice it to say, that she had great pain in the toes, accompanied with a stinging, burning sensation. The limb did not swell much, but the whole foot and ankle soon became discolored, dry, and without smell, looking as black as charcoal. From the ankle upward it had a mottled appearance all over the leg. I also found the temperature of the member much lower than natural. Although this is called dry mortification, in this case there was a separation of the cuticle, and a dark bloody serum effused into vesicles, as in acute mortification, and the bottom of the vesicles were dark colored and livid. The march of the disease was not rapid. In thirty-five days after the parts became affected, all the toes of the right foot dropped off at the second joint. At this stage of the disease it threw off a very disagreeable odor.

Her appetite is good, and she does not seem to waste as much as I had expected. I have described the symptoms and the course of the case up to the time of writing. Her pulse ranges between fifty-five and sixty. My *confrére* was called in; he commenced feeling along the tibial and fibial arteries up to the popliteal space, and finding no ossification of these arteries, asked me upon what did I base my diagnosis. I told him that I recognized a deficiency of the heart's action in the irregular beating of the pulse, and the difficulty she experienced in respiration. Now what are the causes of gangrena senilis? It has been generally supposed that there was ossification of arterial trunks, and sometimes we can feel very distinctly that the artery is ossified. The larger arteries are not invariably ossified in this disease. *Cruveilhier* always referred gangrena senilis to ossification.

There must be some unfavorable circumstance combined with the ossification, as impaired health, disease of the heart, or its valves, producing disorder of the circulation. Then the venous blood can not pass freely, and accumulates in the lower extremities, impeding the circulation. It is doubted by many good surgeons whether ossification of the arteries alone is capable of producing gangrena senilis. I think it is not. Among the causes I would name great universal debility, extreme old age, a thickening and ossification of the coats of the arteries, and a consequent diminution of their capacity, and of their muscular and elastic power.

To the case in question—having become satisfied as to the nature of the case, a good nutritious diet was ordered. I also wrote a prescription for an ointment, composed of the following:

℞ Goulard cerate, - - - ℥ ii.
Ext. conii, - - - - - ℥ ss.
Acidi. carbol. - - - gtt. xxv.
Chloroform, - - - - ℥ ss.
M. S. Apply pro re nata.

This mixture was used to control pain, which it did in a measure, but did not seem to arrest the march of the disease. I also directed her a half grain of *Morphia* three times a day. Under this treatment she seemed to get along comfortably until the 26th of December, 1867. She then complained of a heavy weight over the epigastrium, and costiveness. I ordered her a bottle of the *Citrate of magnesia*, which moved her bowels, but appeared to increase the pain in the stomach, and commenced to vomit every thing she ate. I called to see her on the 28th, and found that nothing would lay on her stomach. I gave the following about four o'clock in the evening:

Bismuth nitratis, - - - gr. 1.
Ext. nucis vomic alcohol, - - gr. 1.
Carbonate magnesia, - - gr. vi.
Sacch. albi —, - - - - gr. xv.
Oleo. mentha piper, - - gtts vi.
M. Sg. Divide in duas partes. Sumat unam statim alteram circa mediam noctem.

This quieted her stomach, relieved her bowels, and she seemed all right. I still continue the ointment, but give her plenty of good brandy. I have omitted the *Morphia*, because we only get the anodyne, but in *Opium* we get an anodyne, a stimulant, and an absorbent. I now have her on one grain and a half of *Opium*, given three times a day.

The 2nd of January, 1868, I took the knife and made five deep incisions, corresponding with the metatarsal bones, in order to give vent to the putrid matter in the cellular tissue. I consider the knife infinitely preferable to the uncertain and tedious method of procuring the detachment of the mortified parts by suppuration; because it diminishes the bulk of the slough, and thereby abates the fœtid effluvia.

January 10th. All of the denuded bones have been removed. I was careful not to injure the living parts. The operation occasioned very little pain, and there was no hæmorrhage of any consequence. I am very confident that surgeons will reprove me for using the knife, but in this case the process of separation was so slow that I became impatient; I therefore departed from an axiom that was always considered by me as false. Besides following a maxim of Celsus, I preferred employing a remedy, though considered by some as uncertain, rather than abandon the patient to an inevitable death—*Satis est enim anceps auxilium experiri quam nullum.* The parts have taken on a healthy appearance, the acute and throbbing pains have subsided; the only sensation now experienced, is that of itching. Pardon me for trespassing on your space, but I attribute the recovery of this case to the prompt use of *Opium* and the *knife.*

